# New Products

**DOI:** 10.1155/S146392468400033X

**Published:** 1984


Journal of Automatic Chemistry, Volume 6, Number 3 (July–September 1984), pages 164–170

### New Products

### Critical point dryer

Balzers's CPD 020 dries biological specimens which might be damaged by the forces resulting from surface tension during normal drying. The critical point method takes advantage of the fact that the phase boundary between liquid and gaseous states disappears when the liquid reaches a certain pressure and temperature. Beyond the critical point the densities of the liquid and the gas are identical.

The drier provides excellent observation of the specimen, having large sight glasses mounted both on the side and top of the specimen chamber. The unit incorporates electronic regulation of the chamber temperature, with automatic pre-cooling with $CO_2$ and heating adjustable up to 50°C. A magnetic stirrer is included and the chamber is certified up to 200 bar. The unit is completely safe in operation.

*A full list of specimen mounts and accessories is available from Balzers at Northbridge Road, Berkhamsted, Hertfordshire HP4 1EN, UK. Tel.: 04427 2181.*

Circle No. 124 on Reader Enquiry Card

### Aggressive liquids

A liquid density transducer with all its wetted parts manufactured from Hastelloy C-276 has been introduced by Solartron Transducers. Designated Type 7841, this transducer provides continuous and accurate measurement of liquid density and is ideally suited to process industry requirements for monitoring aggressive liquids. As with other transducers in the Solartron range, type 7841 is of totally sealed, all-welded construction. It follows the Solartron smooth bore, straight-through flow, vibrating element design. The element containing the flowing liquid is vibrated at its natural resonant frequency, which is measured and maintained by means of a feedback signal. A change in density of the liquid alters the mass of the vibrating system and hence its natural frequency. This measured frequency changes in a direct relationship with the liquid density.

Manufactured with full traceability and to an intrinsically safe design, the 7841 liquid density transducer incorporates a 100 Ω platinum resistance thermometer, which, when used with an associated signal processor, automatically applies a correction factor for the temperature variation effect on the vibrating element. Solartron liquid density transducers are exceptional in that they incorporate a self-compensating feature for variations of pressure up to 30 bar. The signal processor provides a displayed value or 4–20 mA signal of corrected line density, which can be transmitted and processed without any loss in accuracy. Above 30 bar, an online sensor can be used with the signal processor to compensate for the varying pressure effect. The temperature-compensated signal—and pressure compensation where appropriate—can be also used by the signal processor to refer the corrected line density to any selected base condition, i.e. to provide direct readings of referred density or specific gravity. These instruments are thus well suited to mass metering applications where high accuracy is of direct benefit.

*More information from Solartron Transducers (Schlumberger Electronics [UK] Ltd, Victoria Road, Farnborough, Hampshire GU14 7PW, UK. Tel.: 052 544433).*

Circle No. 125 on Reader Enquiry Card

### Automatic sampler

The AOC 8, a pneumatically-operated syringe-type sampler, automates all sampling procedures, syringe washing, sampling of the desired volume and injection. The sampling unit accommodates 50 sample vials with an individual solvent wash for each, or 100 sample vials when the syringe can be washed with the next sample. The controller unit can select the mode of analysis, isothermal or temperature programmed, select the washing agent, set the number of the final sample, set the number of repetitions of analysing one sample, adjust the sample volume, and give instructions to start operation.

To launch Shimadzu's new sampler, Dyson Instruments Ltd are offering a completely automatic system comprising a GC-8APF (dual FID, temperature programmed with automatic cool-down), an AOC-8 sampler, and a C-R3A computing integrator with 176 K of user RAM and built-in printer/plotter for £8600 plus tax.

*Shimadzu chromatographic instruments are sold in the UK by Dyson Instruments Ltd, Sunderland House, Station Road, Hetton, Houghton-le-Spring, Tyne & Wear DH5 0AT, UK. Tel.: 0783 260452.*

Circle No. 126 on Reader Enquiry Card

### Discriminant analysis

Technicon have a new package for identification of raw materials in the pharmaceutical industry. The package consists of two forms of instrumentation: a full-scanning NIR analyser with suitable software, the InfraAlyzer 500; and a filter instrument, the InfraAlyzer 400, with a Hewlett-Packard 85 with full software to be able to discriminate raw materials in the warehouse. It is envisaged that the scanning machine will be used for research, development and laboratory quality assurance, whereas the filter instrument may be used in the warehouse or dispensary.

The main advantages of this package are its simplicity of use and speed of analysis: any operator can obtain a reliable result in a few minutes.

Evaluations have been carried out on a number of raw materials.

*For further details please contact Technicon Instruments Co. Ltd, Evans House, Hamilton Close, Basingstoke, Hampshire RG21 2YE, UK. Tel.: 0256 29181.*

Circle No. 127 on Reader Enquiry Card

### Conductivity meter

An 'all-British', portable conductivity meter has been announced by Datronix Controls. Called the CM21, the meter has been designed and manufactured to provide high accuracy and a wide sensitivity range extending to 200 000 microsiemens for plant, laboratory and field use. The new unit will appeal to industries where monitoring of water or chemical solutions is a requirement. For this reason, calibration is in terms of both microsiemens and parts per million of total dissolved solids.

The linear calibration scale has a width of $3\frac{1}{2}$ in; shows the range 1–10 microsiemens, but is connected to a five-range multiplier control which takes the reading in multiples of 10 to the 10 000–100 000 microsiemens scale. This range is doubled with the special measuring probe with which the new meter is equipped. Total calibration width is $17\frac{3}{4}$ in, which gives accurate readings.

A special hand-held measuring probe has a precisely ground carbon electrode embedded in an epoxy resin base and is shrouded with an individually formed plastic shield, providing the sensor with an accurate measurement environment.

Meter needle movement is of high torque design with controlled damping giving fast deflection with virtually nil overswing.

Automatic temperature compensation is provided at 2%/°C in which all readings are corrected to 25°C. An override button allows direct conductance measurement to be made.

Internal circuitry of the meter relies entirely on the use of integrated and solid state technology: this demands less power and the normal battery life is one year or more. A check button is provided for battery testing.

*Details from the Sales Office, Datronix Controls Ltd, Datronix House, Lower King's Road, Berkhamstead, Hertfordshire HP4 2AE, UK. Tel.: 04427 73377.*

**Circle No. 128 on Reader Enquiry Card**

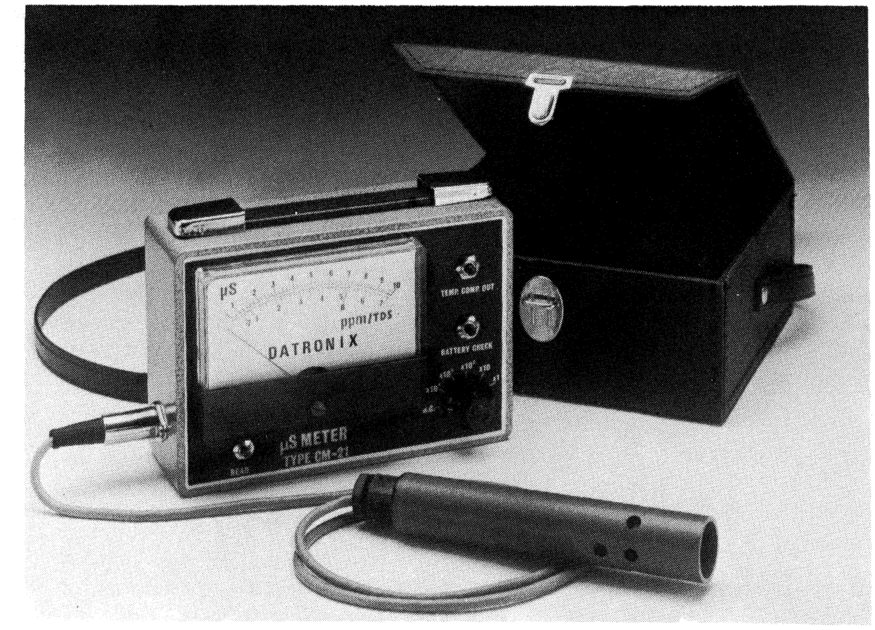

*The CM21 meter from Datronix Controls. It is strongly constructed of diecast aluminium with blue enamelled finish. The meter is provided with an integral handle, weighs 3 lb, and measures $6\frac{3}{4}$ in × 5 in × $2\frac{3}{4}$ in. It is supplied in a black leather-case with shoulder strap. The normal price is £146 (exclusive of tax).*

### RS 232-compatible interface

Comark Electronics have provided their 6600, 6800 and 6020 microprocessor-based thermometers with RS 232-compatible 0–5 V TTL level outputs.

The 6600 incorporates a 10-input automatic scanning system, in addition to such other facilities as variable dwell, pause, alarms, scaling, auto-zeroing and auto-calibrating. It will accept inputs from six types of thermocouples. The 6800 is similar but is for use with PRT inputs.

The 6020 is a portable mains/battery operated microprocessor-based instrument able to accept 10 inputs from K-type thermocouples and to store up to 650 temperatures.

*Detailed leaflets containing full descriptions and specifications are available from Comark Electronics Ltd, Rustington, Littlehampton, West Sussex BN16 3QX, UK. Tel.: 09062 71911.*

**Circle No. 129 on Reader Enquiry Card**

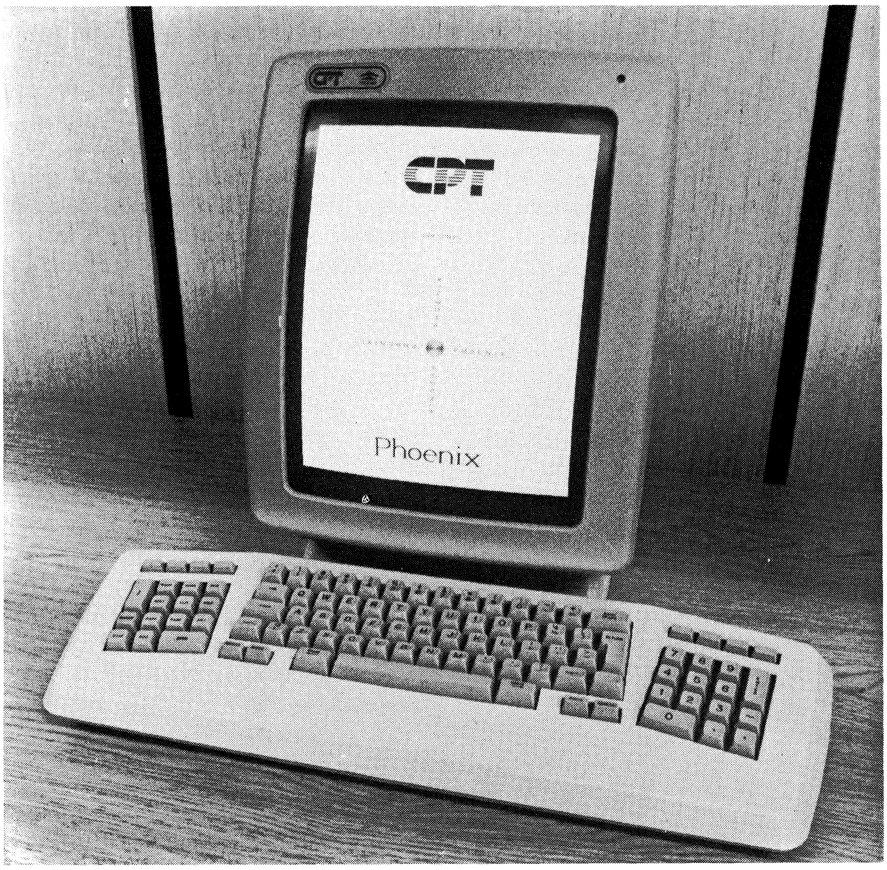

*The CPT Phoenix is claimed to be the World's most powerful word-processor. It combines word-processing capability with graphics (including full scientific and chemical notation), computer-aided design abilities and computerized typesetting. The ergonomic keyboard features reversal of the angled function and numeric pads to suit either left- or right-handed operators. The CPT Phoenix costs £13 200 (excluding tax) and is available from CPT(UK) Ltd, 4th Floor, Trafalgar House, 2 Chalk Hill Road, London W6 8DN. Tel.: 01 741 9050.*

**Circle No. 130 on Reader Enquiry Card**

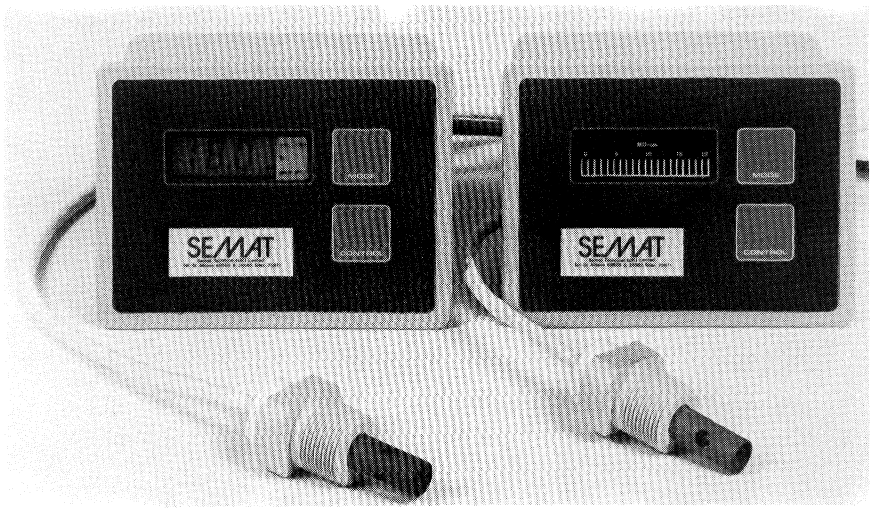

*Thornton Associates, Inc.'s DOT. The resistivity/conductivity meter is available in mains (230 V± 10%) or battery format (4 × 1·2 V). The battery is easily recharged and provides 3 h of continuous running.* (Semat Technical Ltd, UK.)

### Resistivity/conductivity meter

A compact meter measuring resistivity or conductivity for sampling water or for quality-control checks is now available from Semat, the exclusive UK distributor for Thornton Associates, Inc., Massachusetts, USA.

The Thornton DOT is microprocessor based and provides automatic temperature compensation referenced to 25°C. A precision RTD sensor in an 0·1 constant cell allows accurate readings to ± 1%. Cells can be used for dip or in-line process monitoring. Temperature readout (0–100°C) is standard on the LCD, besides the 0–20 MΩ-cm or 0–2500 μS-cm conductivity providing, in effect, a dual-purpose instrument. The watertight NEMA 4X enclosure allows protection for the electronics against water and chemicals, such as acids or etchants, used in electronic manufacture and processing. The meter can be used on distilled water supplies and finds widespread use in the medical, clinical and pharmaceutical fields. For efficient operation of purification equipment, resistivity/conductivity and temperature are two of the most important quality parameters requiring accurate measurement.

Compactness (5 in × 4 in × $2\frac{1}{2}$ in), and ease of installation, allows fitting to any wet bench and with a two-cell input capability allows control of outgoing rinse water and incoming DI water quality.

*Full details, literature, prices and technical back-up are available from Semat Technical Ltd, 223 Hatfield Road, St Albans, Hertfordshire AL1 4UN, UK. Tel.: 0727 34585.*

**Circle No. 131 on Reader Enquiry Card**

### Optical gratings

Spring 1985 will see the launch of Hilger s full range of plane and concave gratings for spectroscopic work. Whilst Hilger holographic gratings have been in production since 1970 they have been largely reserved for use in the company's own products. However, a substantial increase in production capacity and a widening of the available range enables Hilger Analytical to offer these to the general market. Hilger Analytical has been involved in spectroscopy throughout the whole of this century and claim to be one of the most experienced in the field. Glass, quartz and alkali halide prisms were the main dispersive elements produced by Hilger during the earlier decades. The company then became one of the first in the World to have its own ruling machine, from which quantities of precisely ruled master gratings were produced. When the holographic technique became known, Hilger was again one of the first to employ it for full-scale production.

The new extended range of plane and concave gratings being offered are true masters, not replicas. The result is a series dispersing media suitable for use in the best spectrometer and monochromator systems.

Aperture sizes range from 25 mm diameter to 110 mm square. Frequencies vay from 600 to 3600 lines/mm, all optimized for a specific wavelength range. Concave gratings are available in a variety of different focal lengths. Work is currently proceeding on the introduction of blazed holographic gratings.

These alternatives make up the standard range—effectively to be made available off-the-shelf. However, Hilger Analytical will consider special requirements and, if practical, will produce gratings with other specified frequencies.

*For further information contact Jack Reed, Crystal Materials Group Manager, Hilger Analytical Ltd, Westwood, Margate, Kent CT9 4JL, UK. Tel.: 0843 25131.*

**Circle No. 132 on Reader Enquiry Card**

### Literature for chromatographs

Beckman's journal *Chromatogram* has a new format and will be published at regular intervals. It is a publication which includes information on HPLC techniques and reviews by laboratory users of various types of equipment in the HPLC field.

Beckman is compiling a regular circulation list of people who would like a free copy. Anyone who wants to be sent *Chromatogram* should contact either Gill Johns or Sally Biggs at Beckman's High Wycombe headquarters (tel.: 0494 41181) or fill in the appropriate number on *JAC*'s reader enquiry service card. Beckman's Applications Chemist, Martin Parker, is also interested in receiving contributions on HPLC techniques for publication in *Chromatogram*; potential authors should contact him directly by telephone at the number above or write to him at Beckman's High Wycombe address (Progress Road, Sands Industrial Estate, High Wycombe, Buckinghamshire, UK).

**Circle No. 133 on Reader Enquiry Card**

### LC detector/data system

The PU 4021 multichannel UV detector can store up to nine UV/Vis spectra, and the addition of a new data station permits hundreds of spectra to be stored in a single chromatographic run. The PU 4850's software features full QWERTY keyboard control and a sophisticated display program for post-run spectrum manipulation. Even in this configuration, the system allows four-channel integration and full control of LC instrumentation.

There are two modes of operation. The chromascan mode enables storage of complete UV/Vis spectra at a programmable rate to a maximum of one per second. An on-line VDU displays the results as a real-time three-dimensional view (or chromascan, see photograph) of the sample. This may be displayed from a number of different viewpoints ensuring that no data is hidden. Furthermore, any data in the spectral or chromatographic

(time) domains is selectable for further manipulation and integration.

The second mode of use involves the selective storage of spectra from a chromatographic run. It is possible to set parameters that will enable a spectrum to be acquired at a peak apex, at inflection points, at a pre-set retention time, or manually at any time. These peak detection algorithms may be run concurrently, providing the data required for peak authentication and for monitoring peak purity.

Spectra which have been acquired can be viewed using a program that displays spectra singly, or up to four at a time. For direct comparison of spectra the superimpose, or normalize and superimpose options, may be chosen. Where the differences in spectra are subtle, comparison is further aided by first and second derivative displays. Spectrum subtraction, and normalize and subtract routines, facilitate comparison of reference spectra with complex spectra containing two or more components. Spectrum addition enhances signal-to-noise ratios where the original spectra are very weak.

*Information from Pye Unicam Ltd, York Street, Cambridge CB1 2PX, UK. Tel.: 0223 358866.*

Circle No. 134 on Reader Enquiry Card

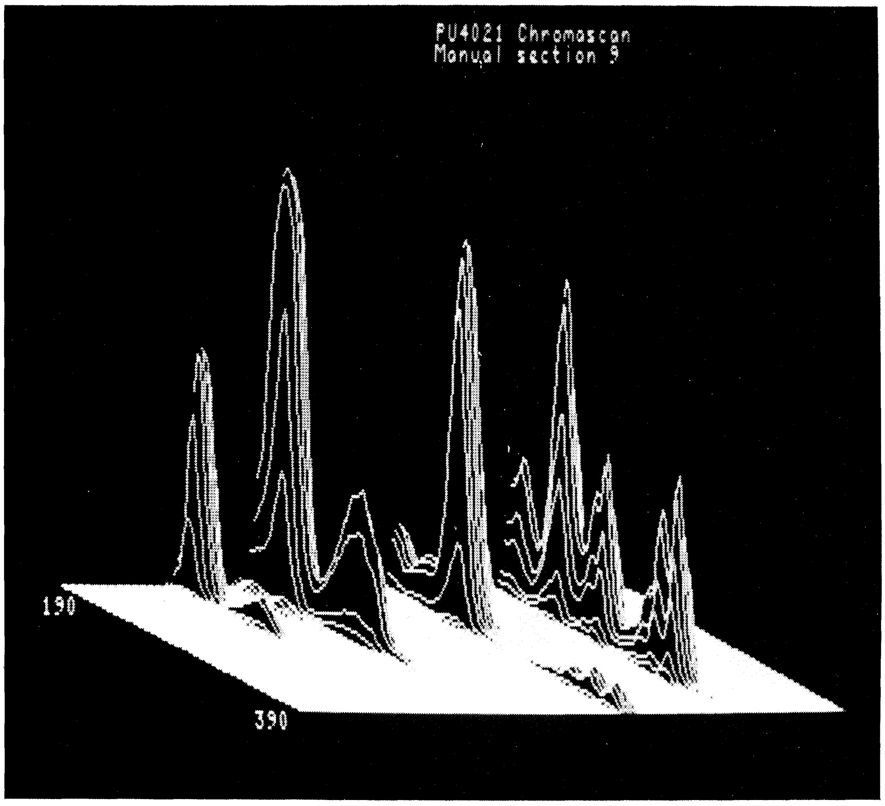

*A chromascan (a real-time three-dimensional display) generated by Philips's PU 4850 data station. The disk drive system provides for storage of fully annotated spectra and chromatograms; so the analyst can build up a personal library of reference spectra and standard chromatograms, which can be accessed at any time, to compare with current samples.*

*This new computerized gas chromatograph from Shimadzu, the GC-9AM, has all the features of the well-known 'thinking' GC-9A, but utilizes a unique and fully patented modular system in the analytical section. The sample-injection port, column, and detector are combined into one modular analysis unit to facilitate quick change of analytical conditions. These analysis units are easy to mount and dismount, and up to four analysis units can be installed together. Provision of extra analysis units eliminates cross-contamination between samples and significantly reduces down-time when one of the detectors has to be replaced. When connected to the Shimadzu C-R3 A Chromatopac and automatic sampler (AOC-9) the GC-9AM can analyse mixtures, measure results and judge them against standards or expectation. If the results vary, the instrument can automatically reanalyse under new analytical conditions until the results are improved or confirmed to be at variance. Details from Dyson Instruments Ltd, see p. 164.*

Circle No. 135 on Reader Enquiry Card

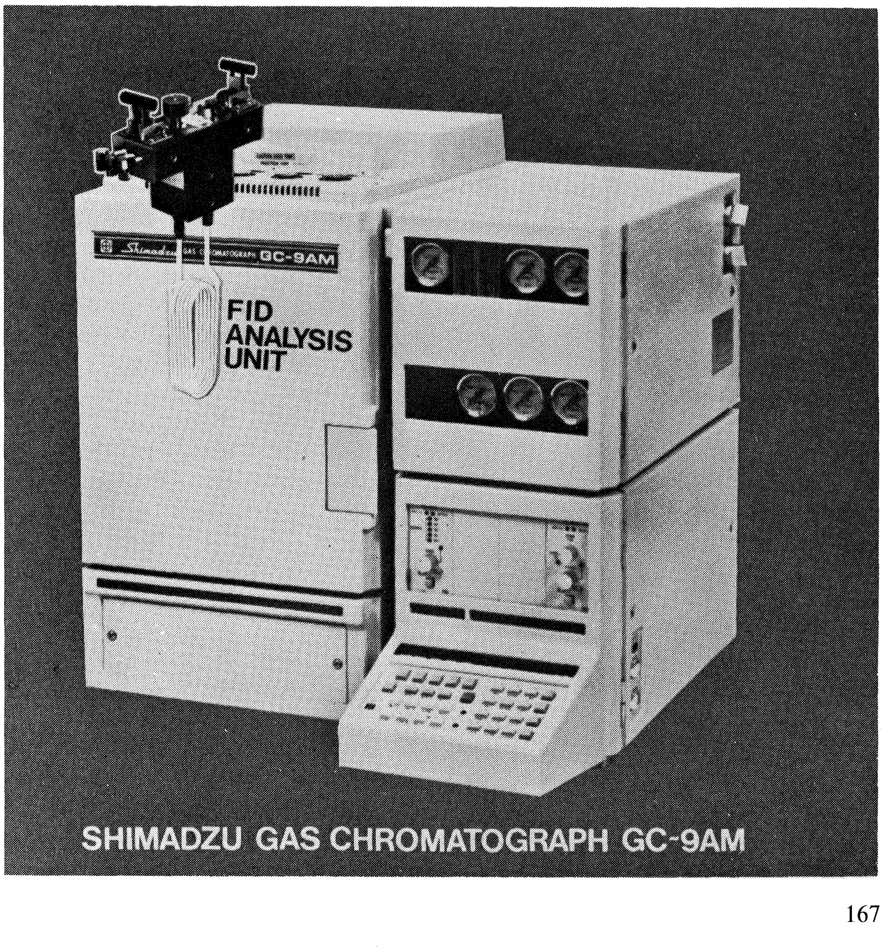

### Zeeman/3030

Stabilized temperature platform furnace (STPF) atomic absorption spectroscopy (AAS) with Zeeman background correction is now recognized as the best technique for determining most metals at ultra-trace concentration levels. The STPF conditions virtually eliminate chemical interferences and Zeeman background correction overcomes high signal attenuations and spectral interferences caused by the matrix; so it is possible to directly analyse samples that previously required solvent extraction or the method of additions.

Perkin-Elmer have a new system dedicated to STPF atomic absorption spectroscopy: the Zeeman/3030, which consists of a Model 3030 atomic absorption spectrophotometer, an HGA-600 graphite furnace (with Zeeman-effect background corrector), and an AS-60 autosampler. It is simple to operate and analytical methods can be developed using the high-speed graphics display and data handling.

In graphite furnace AAS, the sample matrix produces non-atomic signals that can be compensated for by using a background corrector. The a.c. Zeeman background corrector in the system allows correction of background signals of over 2 Absorbance units. So the instrument is useful for determining very low concentrations of elements in complex matrices with high salt or total solids content. Since structured background is always corrected for directly on the analyte line, the Zeeman/3030 gives good results when the sample produces background absorption with a fine spectral structure.

Under some circumstances, chemical interferences cause severe analytical errors in graphite furnace AAS. In the HGA-600, samples are atomized into an environment that has almost reached thermal equilibrium, so that vapour-phase interferences are eliminated.

Method development is fast. Both the analyte and background signals are numerically and graphically displayed so the user can see how changes of parameters affect signal height and shape. Analytical parameters for user-developed and standard methods are stored on floppy disk and can be retrieved to set up the instrument automatically. Each disk holds 30 methods and any number of disks can be used, giving unlimited methods of storage.

The Zeeman/3030 is calibrated by the AS-60 autosampler, which makes up standards and uses up to eight of them for each calibration. Matrix modification, necessary for determining many elements accurately, is also automated. The AS-60 adds one or two matrix modifiers alternately or simultaneously to remove the matrix efficiently and aid analyte atomization.

*For further information contact Perkin-Elmer Ltd, Post Office Lane, Beaconsfield, Buckinghamshire HP9 1QA, UK. Tel.: 04946 6161.*

**Circle No. 136 on Reader Enquiry Card**

### More Perkin-Elmer

### *LCI-100 Laboratory Computing Integrator*

The Model LCI-100 is a compact, single-channel instrument for gas or liquid chromatography. It acquires data at high speed—up to 100 points/s—reduces the data and reports the results.

Software includes replot and reintegrate functions for method development. The last chromatogram is always stored, so that corrections can be made without rerunning the sample. Multiple methods can be stored, saving set-up time between analyses, and any method in memory can be used for reintegration. Multiple peak files can also be saved, so that calibration runs can be averaged in a single operation. The LCI-100 provides all standard chromatography calculations, based on peak height and/or peak area. Autosequencing software facilitates use with autosamplers and includes method chaining. Summary reports provide additional information on the chromatogram, for example the number of unidentified peaks and the total peak area.

The LCI-100 is designed around the Motorola MC68000 and offers a total of 384K bytes of memory (256K bytes of ROM and 128K bytes of RAM). Its full alphanumeric keyboard, with special function keys for chromatography and liquid crystal display, simplify operation. A high-speed $11\frac{1}{4}$in printer/plotter is part of the system.

The LCI-100 can be started from an external device. Six external event relays and BCD input from autosamplers are available as accessories and optional RS232C or IEEE-288 interfaces allow the system to communicate with other devices.

### *Thick-film bonded phase columns*

Thick-film bonded phase fused silica capillary columns, now available from Perkin-Elmer, are particularly useful for applications that require increased sample capacity: trace analysis, on-column injection and the GC-MS and GC-FTIR techniques for example.

Cross-linking of the stationary phase in the new columns increases the stability of the liquid phase and offers several benefits. The operating temperature is extended, which gives better resolution and separation in the subambient region and reduces column bleed at higher temperatures. Coatings 5 microns thick give increased loading capacity and allow better separation resolution of low molecular weight components. More consistent performance from column to column is achieved due to close monitoring of the chemical composition of the liquid phase and the increased inertness of the flexible silica tubing.

The columns are offered in two lengths—25 M and 50 M—and two different liquid phases, methyl silicone (equivalent to OV-1) and methyl 5% silicone (equivalent to SE-54).

*Details from Perkin-Elmer (above).*

**Circle No. 137 on Reader Enquiry Card**

### Potassium channel for ICP spectrometer

A special detector assembly for potassium is now available for use with the Philips PV8350 ICP emission spectrometer. The PV8390 potassium detector assembly is the third unit for alkali elements to be developed by Philips; devices for sodium (588·9 nm) and lithium (610·3 nm) are already available.

The best lines for measuring the majority of elements by ICP emission spectrometry are grouped at shorter wavelengths than 400 nm. The alkali elements are the most notable exceptions to this rule: their most sensitive lines are towards the longer wavelength side of the visible spectrum. It is possible to extend the wavelength range of a vacuum-emission spectrometer to include the alkali elements by fitting a grating with fewer lines per mm, thereby bringing the longer wavelengths to the position of the normal camera. A disadvantage is that dispersion is sacrificed and the shortest wavelengths can no longer be measured in the first order of the grating. Major light losses are inevitable due to the interference filters needed to remove the overlapping first-order spectrum. This has been overcome in the PV8350 by means of special detector assemblies for sodium and lithium which operate with the 2160 lines/mm holographic grating. Potassium can also now be added to such a system. In the PV8390, plasma light focused on the end of an optical fibre is guided to a narrow band K 766·4 nm interference filter and measured by a red-sensitive photomultiplier. For stability, these optical components are housed within the temperature-controlled enclosure of the spectrometer.

Among the applications of this new accessory is the determination of potassium in slags in the analytical programs of a PV8350 ICP spectrometer delivered to the steel industry.

*Philips ICP emission spectrometry systems are marketed in the UK by Pye Unicam Ltd, York Street, Cambridge CB1 2PX, UK. Tel.: 0223 358866.*

Circle No. 138 on Reader Enquiry Card

### Peptide synthesizer

The Model 430A peptide synthesizer, a system for fully automated, high-efficiency synthesis, was announced by Applied Biosystems this Spring. The system should enable researchers with little or no prior experience to synthesize peptides in their own laboratories. These researchers previously have purchased peptides at very high cost with long turn-around times and, frequently, at purity levels that forced them to go through difficult purification procedures.

The key to the very high coupling yield of the Model 430A is a novel preactivation system which converts each amino-acid to a very efficient reacting species immediately prior to each coupling step. To further assure high coupling yields, Applied Biosystems also manufactures and supplies all the necessary synthesis reagents.

The system features a defined protocol optimized for each amino-acid. It can also be fully programmed by the user for straightforward adaptation to other chemistries. This combination of a preactivation system and flexible programming allows experienced peptide chemists to easily implement a variety of automated chemistry modifications and synthesis alternatives.

*For more information contact Bernard Herd, Applied Biosystems GmbH, Bergstrasse 104, D6102 Pfungstadt, FR Germany; tel.: 06157 6036; or Applied Biosystems, Inc., 850 Lincoln Centre Drive, Foster City, California 94404, USA; tel.: 415 570 6667.*

Circle No. 139 on Reader Enquiry Card

### Sequential X-ray spectrometer

Siemens's microprocessor-based SRS 300 has a rhodium end-window tube, which gives an improvement in performance compared to side-window tubes. The rhodium tube emits strong L X-rays, as well as Bremsstrahlung, and is suited for analysis of heavy and light elements.

The rhodium end-window tube can be used with all chemical elements without having to change tubes. It improves intensity, measuring time and accuracy; both boron and carbon can be analysed qualitatively and quantitatively. Suitable excitation conditions (high voltage and current) can be set in routine operation. These advantages are obtained by having the anode at a higher potential. The window tube is, therefore, not subject to thermal load owing to backscatter electrodes.

The tube has a window only 125 mm thick and is consequently suitable for long-wave L X-rays. The end-window tube is also sufficiently large for the sample to be well illuminated. The entire X-ray radiation is used to excite the sample and carry out analysis. Intensity is also improved by having the radiation entering vertically through the end-window tube (lower absorption).

*More information from Siemens Ltd, Siemens House, Windmill Road, Sunbury-on-Thames, Middlesex TW16 7HS, UK. Tel.: 09327 85691.*

Circle No. 140 on Reader Enquiry Card

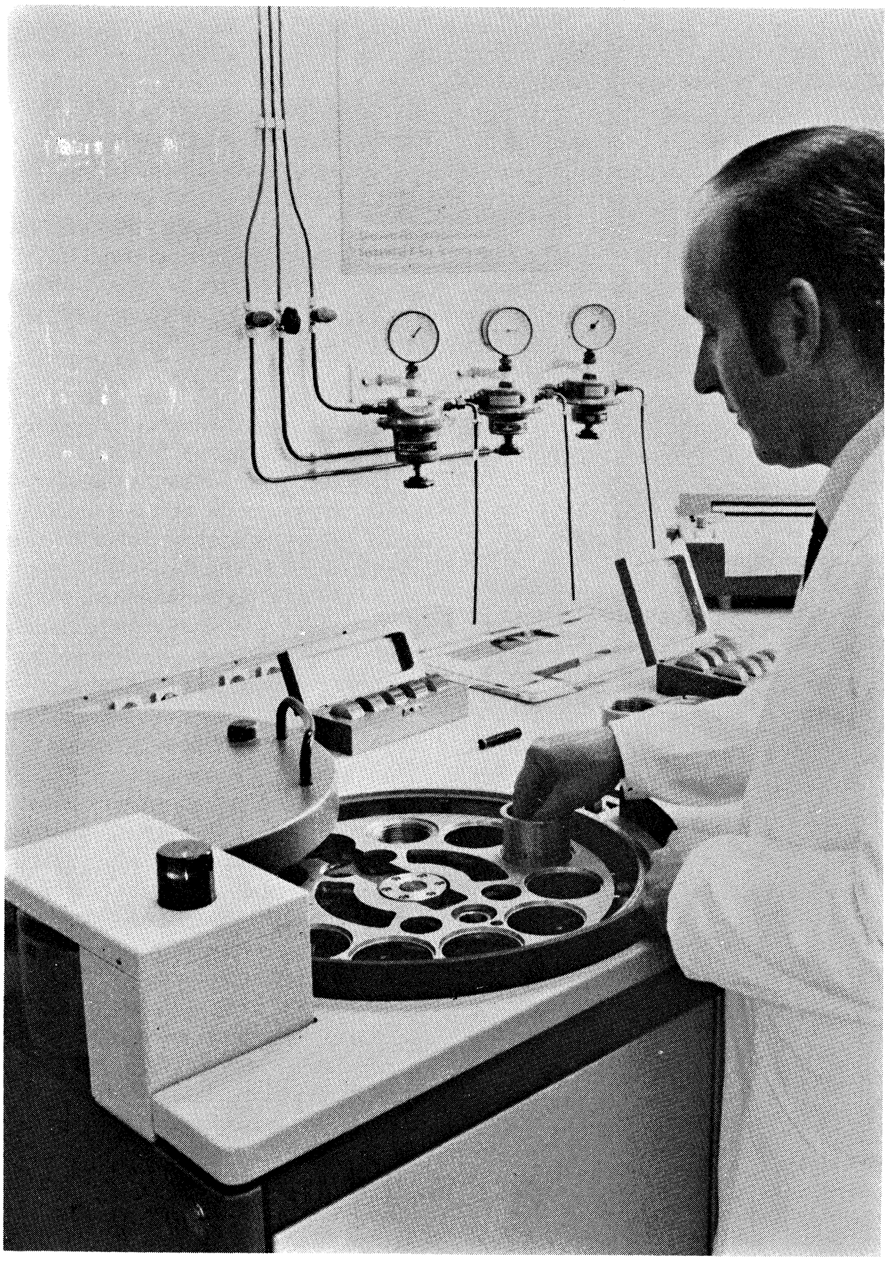

*The SRS 300 microprocessor-based sequential X-ray spectrometer from Siemens, fitted with a rhodium end-window tube. The end-window tube is arranged fully symmetrically so that run-in effects are avoided when operating values are changed. The tube function is restabilized immediately after changes in current or voltage. Delays or inaccurate measurements are consequently avoided.*

### Bench-top GC/MS

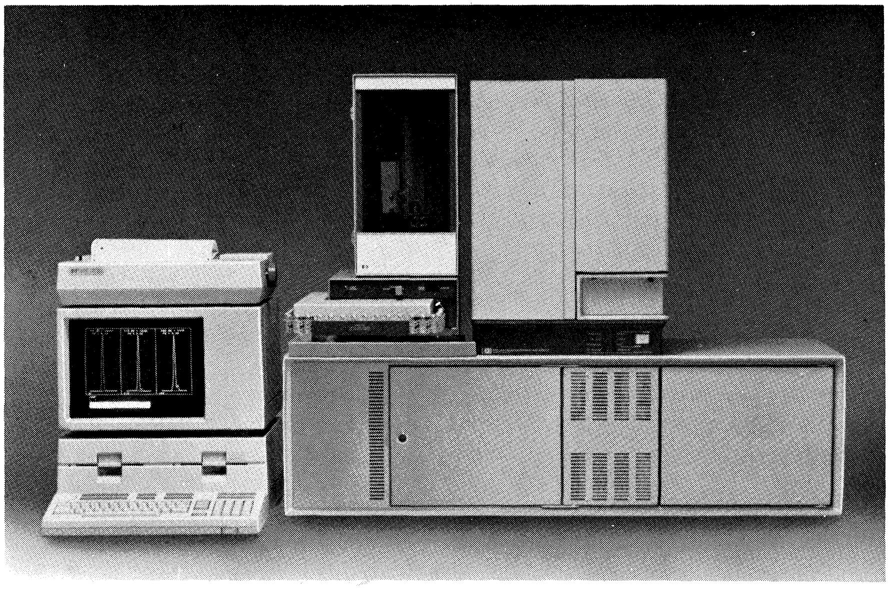

*The HP 5995C bench-top GC/MS features a new GC/MS workstation that simplifies operation, provides sophisticated data handling and full automation. It is shown here with full-colour GC/MS workstation and the optional HP 7672 automatic sampler that permits unattended injection of up to 99 samples. (The HP 5995 series, introduced by Hewlett-Packard in 1979, was the first to offer the chromatographer a bench-top GC/MS at a moderate price. HP 5995s are widely used in chromatography laboratories to help identify compounds in applications such as food, drug, pesticide and petrochemical analysis.)*

The HP 5995C differs from other HP 5995s in that it has a high-performance GC/MS work-station providing data-handling and automation and an interactive CRT. When a method is built, the CRT presents a form. The operator follows the cursor and fills in the blanks; softkeys permit single-keystroke commands. User-built methods automatically can control the entire system, including an optional HP 7672 automatic sampler, in unattended analysis of up to 99 samples from injection through final report. Other system features, such as AUTOTUNE, diagnostics and automatic library search, help simplify operation.

*Analysing and editing GC/MS data*

Interactive data editing provides capabilities previously available only on more expensive systems. After a run, using the data-editing features, data can be manipulated and displayed in a vareity of ways that help identify unknowns and enhance presentation. For example, a portion of a total ion chromatogram can be expanded instantly and displayed in a window on the CRT. The user can overlay spectra and subtract one spectrum from another. Any data on the CRT can be sent to the printer for a hard copy.

*Choice of report formats*

There are three standard report formats to choose from: short, long or extended. In addition, if a custom presentation is needed, VisiCalc can be used. With its built-in RS-232 and HP-IB interfaces and optional terminal-emulation capability, the GC/MS workstation is a gateway to a minicomputer for further data-handling or storage.

*Personal computer*

When it's not doing GC/MS, the workstation can serve as a high-performance HP9000 Series 200 technical personal computer that has over 70 software packages.

*Reader enquiries to Enquiry Section, Hewlett-Packard Ltd, Eskdale Road, Wokingham, Berkshire RG11 5DZ, UK.*

**Circle No. 141 on Reader Enquiry Card**

### PNA analyser

An advanced PNA Analyser has been launched by United Technologies Packard: the Model 412 Piano Analyser is dedicated to the separation of naphtha-type samples by gas chromatography. It replaces the Packard Model 411, which has been the only commercially available PNA Analyser since 1970.

The 412 greatly expands the analytical capabilities of the previous model by means of a new analytical method that allows naphthas with final boiling-points of up to 275°C to be separated. It also adds microprocessor control, a totally new trap, upgraded valves and new operating system. Its analytical module contains three columns: one preseparates saturates and aromatics: the second is a specially treated molecular sieve 13X for separating paraffins and naphthenes by carbon number: the third analyses aromatic compounds according to carbon number and groups the polynaphthenes together (compounds with final boiling-points above 200°C are also analysed on the third column).

Packard's triple column technique surpasses the performance of naptha analysers using only a single capillary column. Model 412 is based on the principle of chromatographic group separation by carbon number in which every single component automatically finds its place in the chromatogram. The separate identification of single components by capillary column analysis is, therefore, unnecessary.

Packard offers three specific options with the new instruments—the PNA option, which separates paraffins, naphthenes and aromatics, the nPiPNA option, which analyses n-paraffins, isoparaffins, naphthenes and aromatics and, the PONA option, which covers paraffins, naphthenes, aromatics and olefins.

The software is specifically designed for analysis. The information facility comprises a CRT display and a keyboard. Instructions are organized into pave formats where the associated parameters for naphtha analyses are already established. Moreover, the software contains 50 different run-time variables, a variety of standard GC control pages and three standard methods for permanent memory storage.

A comprehensive status report, which gives both the set-point data and actual conditions of the instrument, is obtained by the touch of a button. Parameters specific to the various analyses are included in the report: trap temperatures etc.

Packard has pioneered the development of gas chromatography equipment for the automatic analysis of naphtha-type samples. The Model 411 was developed under license from Shell Laboratories 14 years ago.

*Details from Packard Instrument Ltd, 13–17 Church Road, Caversham, Berkshire RG4 7AA, UK. Tel.: 0734 478234.*

**Circle No. 142 on Reader Enquiry Card**

